# ﻿Filling a zoogeographical gap in China: Taxonomic descriptions of six new spider species of the *Pholcusphungiformes* species group (Araneae, Pholcidae)

**DOI:** 10.3897/zookeys.1232.145622

**Published:** 2025-03-19

**Authors:** Jinglin Li, Qiaoqiao He, Shuqiang Li, Zhiyuan Yao

**Affiliations:** 1 College of Life Science, Shenyang Normal University, Shenyang 110034, Liaoning, China Shenyang Normal University Shenyang China; 2 Institute of Zoology, Chinese Academy of Sciences, Beijing 100101, China Institute of Zoology, Chinese Academy of sciences Beijing China

**Keywords:** Biodiversity, cellar spiders, DNA barcode, invertebrate, morphology, new species, taxonomy

## Abstract

The spiders of the *Pholcusphungiformes* species group in China are distributed across the Lüliang Mountains and the Yanshan-Taihang Mountains in northern China, and the Changbai Mountains, which border northeastern China and North Korea. This study presents the first collection of the *P.phungiformes* species group from mountainous regions situated between the Yanshan-Taihang and Changbai Mountains, revealing six new species: *Pholcuschaoyang* S. Li & Yao, **sp. nov.**, *P.hebei* S. Li & Yao, **sp. nov.**, *P.huludao* S. Li & Yao, **sp. nov.**, *P.jinzhou* S. Li & Yao, **sp. nov.**, *P.liaoning* S. Li & Yao, **sp. nov.**, and *P.qin* S. Li & Yao, **sp. nov.** Detailed diagnoses, descriptions, photomicroscopy images, and DNA barcodes of new species are provided.

## ﻿Introduction

*Pholcus* Walckenaer, 1805 is the most diverse genus of the family Pholcidae C.L. Koch, 1850 and is mainly distributed in the Afrotropical, Palaearctic, Indo-Malayan, and Australasian regions (e.g., Huber 2011; Yao and Li 2012, 2025; WSC 2025). This genus comprises 21 species groups and 411 species (Huber 2011; Huber et al. 2018; WSC 2025). The *Pholcusphungiformes* species group is the most speciose with 125 species (e.g., Huber 2011; Wang et al. 2020; Yao et al. 2021; Lu et al. 2022; Zhao et al. 2023a, b; Lee et al. 2024). Almost all of the species in this group have been recorded from four mountain ranges: the Lüliang Mountains (9 spp.) and the Yanshan-Taihang Mountains (35 spp.) in North China, the Changbai Mountains (28 spp.) at the border between northeastern China and North Korea, and the Taebaek-Sobaek Mountains (53 spp.) on the Korean Peninsula. The only exception is *P.phungiformes* Oliger, 1983, which is known from the Maritime Territory, Sakhalin Island, and the Kurile Islands, Russia (Huber 2011).

The records from the Lüliang Mountains represent the westernmost distribution limit of the *P.phungiformes* species group. Furthermore, this group is only distributed north of the Qinling Mountains. These conclusions are supported by the fact that Yao et al. did not find any specimens of this group during their sampling in Shaanxi Province in 2013, 2016, and 2019, nor was any found during a 2022 expedition in the Qinling Mountains, whose range extends from the southern part of Shaanxi Province to the western part of Henan Province (Zhao et al. 2023b; Yang et al. 2024a, b).

**Figure 1.**
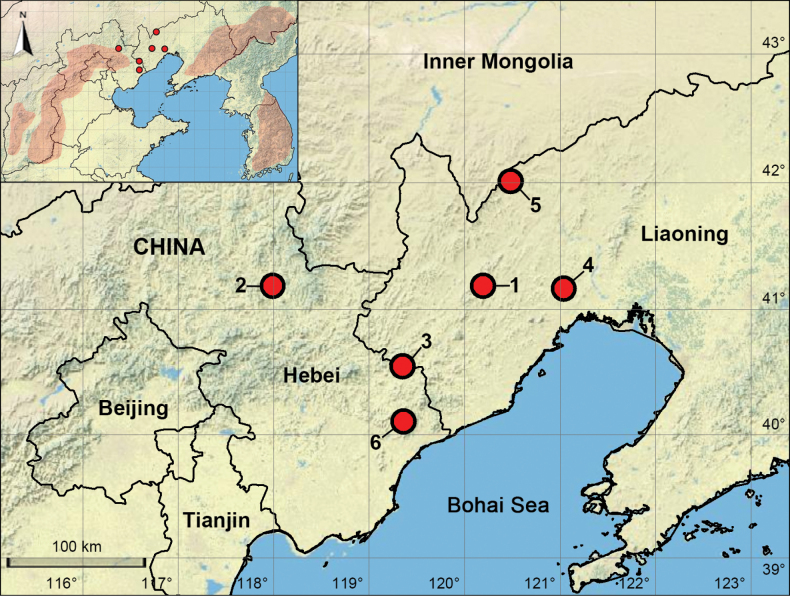
Distribution records of new species of the *Pholcusphungiformes* species group. 1. *P.chaoyang* sp. nov. 2. *P.hebei* sp. nov. 3. *P.huludao* sp. nov. 4. *P.jinzhou* sp. nov. 5. *P.liaoning* sp. nov. 6. *P.qin* sp. nov. Red shading from left to right in the insert indicates the Lüliang Mountains, the Yanshan-Taihang Mountains, the Changbai Mountains, and the Taebaek-Sobaek Mountains.

**Figure 2.**
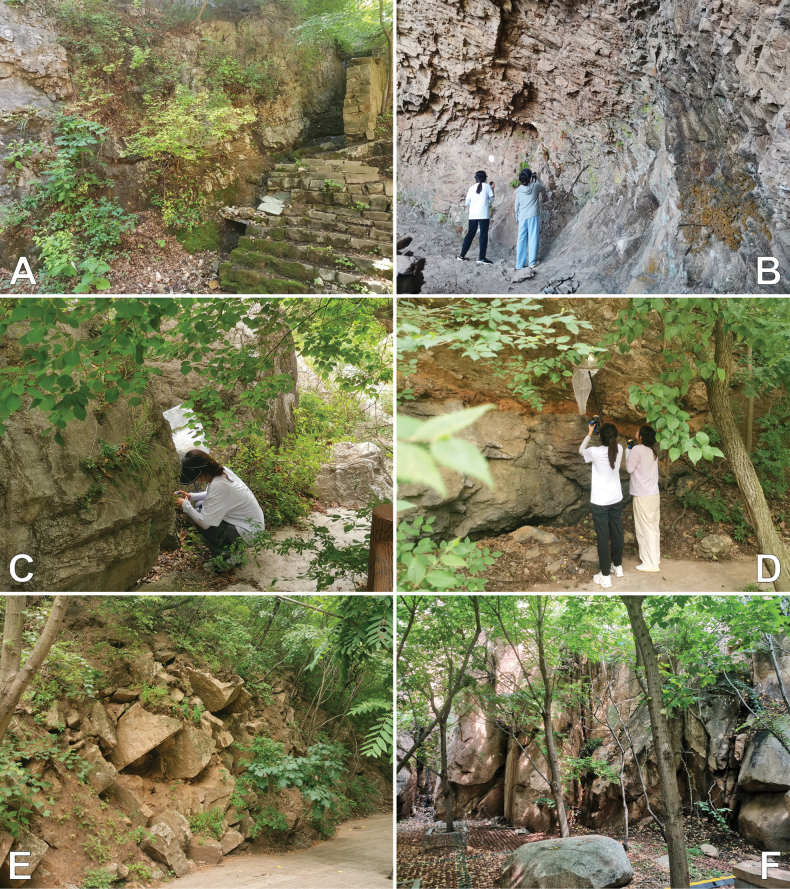
Typical habitats of the new species of the *Pholcusphungiformes* species group. **A** Qingfengling (type locality of *P.chaoyang* sp. nov.) **B** Longfengdong (type locality of *P.hebei* sp. nov.) **C** Longtan Grand Canyon (type locality of *P.huludao* sp. nov.) **D** Beiputuoshan (type locality of *P.jinzhou* sp. nov.) **E** Daheishan (type locality of *P.liaoning* sp. nov.) **F** Bingtangyu (type locality of *P.qin* sp. nov.).

In China, 71 species of the *P.phungiformes* species group have been recorded, accounting for 57% of the global total for this group. Nevertheless, the distribution of this group within China is noticeably patchy, due to the lack of surveys targeting this group in mountainous regions situated between the Yanshan-Taihang and Changbai Mountains. For this reason, in 2024, we conducted a survey in these regions for the first time and report herein six new species (Figs 1, 2).

## ﻿Material and methods

Specimens were examined and measured with a Leica M205 C stereomicroscope. Left male palps were photographed. Epigynes were photographed before dissection. Vulvae were photographed after treatment in a warm 10% solution of potassium hydroxide (KOH) to dissolve soft tissues. Images were captured with a Canon EOS 750D wide zoom digital camera (24.2 megapixels) mounted on the stereomicroscope mentioned above and assembled using Helicon Focus v. 3.10.3 image stacking software (Khmelik et al. 2005). All measurements are given in millimeters (mm). Leg measurements are shown as: total length (femur, patella, tibia, metatarsus, tarsus). Leg segments were measured on their dorsal side. The distribution map was generated with ArcGIS v. 10.2 (ESRI Inc.). The specimens studied are preserved in 75% ethanol and are deposited in the College of Life Science, Shenyang Normal University (SYNU), Liaoning, China.

Terminology and taxonomic descriptions follow Huber (2011) and Yao et al. (2015, 2021). The following abbreviations are used:

**aa** anterior arch,

**ALE** anterior lateral eye,

**AME** anterior median eye,

**b** bulb,

**da** distal apophysis,

**e** embolus,

**fa** frontal apophysis,

**kn** knob,

**L/d** length/diameter ratio,

**pa** proximo-lateral apophysis,

**PME** posterior median eye,

**pp** pore plate,

**pr** procursus,

**u** uncus.

DNA barcode sequences of five new species were obtained. A partial fragment of the mitochondrial cytochrome *c* oxidase subunit I (COI) gene was targeted using the following primers: forward: LCO1490 (5’-GGTCAACAAATCATAAAGATATTGG-3’) and reverse: HCO2198 (5’-TAAACTTCAGGGTGACCAAAAAATCA-3’) (Folmer et al. 1994). Additional information on extraction, amplification and sequencing procedures is provided in Yao et al. (2016). Sequences are deposited in GenBank (accession numbers provided below, Table 1).

**Table 1.** Voucher specimen information.

New species	Voucher code	GenBank accession number	Sequence length	Collection locality
*P.chaoyang* sp. nov.	W354	PV056134	634 bp	Liaoning, Chaoyang, Chaoyang County
*P.hebei* sp. nov.	W358	PV056135	634 bp	Hebei, Chengde, Longhua County
*P.huludao* sp. nov.	W356	N/A	N/A	Liaoning, Huludao, Jianchang County
*P.jinzhou* sp. nov.	W350	PV056132	634 bp	Liaoning, Jinzhou, Guta District
*P.liaoning* sp. nov.	W352	PV056133	634 bp	Liaoning, Chaoyang, Beipiao
*P.qin* sp. nov.	W359	PV056136	634 bp	Hebei, Qinhuangdao, Funing District

## ﻿Taxonomy

### ﻿Family Pholcidae C.L. Koch, 1850

**Subfamily Pholcinae C.L. Koch, 1850**

#### ﻿Genus Pholcus

Taxon classificationAnimaliaAraneaePholcidae

Walckenaer, 1805

555E5210-9983-5C59-B901-B52CD6147CE4

##### Type species.

*Araneaphalangioides* Fuesslin, 1775.

### ﻿*Pholcusphungiformes* species group

This species group was recognized by Huber (2011). The six new species described below are assigned to this group by the following combination of characters: male chelicerae with frontal apophyses, male palpal tibia with a prolatero-ventral projection, uncus with a “pseudo-appendix”, and epigyne with a knob.

#### ﻿ Pholcus chaoyang

Taxon classificationAnimaliaAraneaePholcidae

S. Li & Yao sp. nov.

29DC42C0-559A-5DFB-9D51-CD9777AF8DE1

https://zoobank.org/404BD282-8958-457B-B7B9-B699C681BDFC

Figs 3 , 4

##### Type material.

***Holotype***: China • ♂; Liaoning, Chaoyang, Chaoyang County, Qingfengling Town, Qingfengling Scenic Spot; 41.186698°N, 120.190401°E; alt. 486 m; 17 Jul. 2024; Z. Yao, J. Li & M. Yan leg.; SYNU-Ar00415. ***Paratypes***: China • 1♂; same data as for the holotype; SYNU-Ar00416 • 2♀; same data as for the holotype; SYNU-Ar00417–18.

##### Etymology.

The specific name refers to the type locality; noun in apposition.

##### Diagnosis.

**Figure 3.**
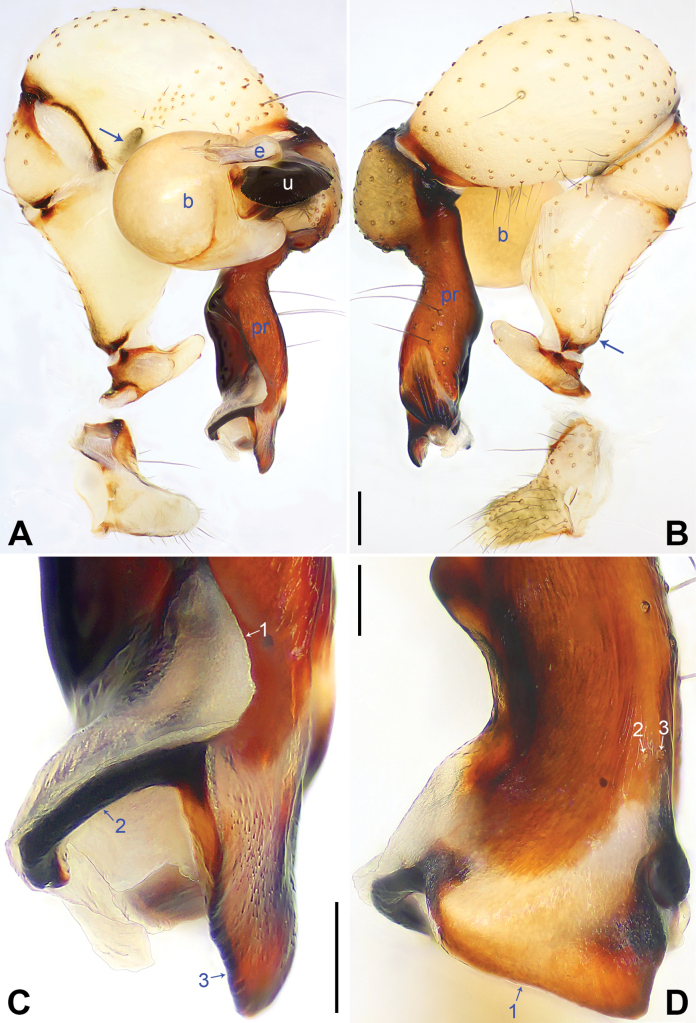
*Pholcuschaoyang* sp. nov., holotype male. **A, B** Palp (**A** prolateral view, arrow points at prolatero-ventral protrusion **B** retrolateral view, arrow points at retrolatero-proximal protrusion) **C, D** distal part of procursus (**C** prolateral view, arrow 1 points at prolatero-subdistal membranous edge, arrow 2 points at sclerotized apophysis, arrow 3 points at distal apophysis **D** dorsal view, arrow 1 points at distal edge, arrows 2, 3 point at dorsal spines). Abbreviations: b = bulb, e = embolus, pr = procursus, u = uncus. Scale bars: 0.20 mm (**A, B**); 0.10 mm (**C, D**).

**Figure 4.**
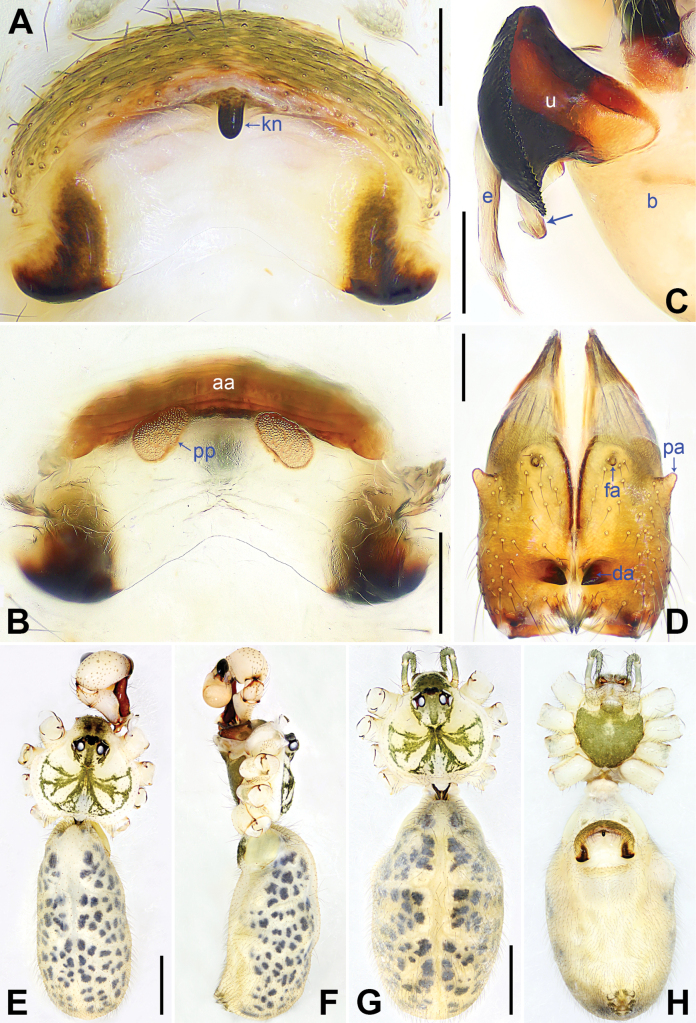
*Pholcuschaoyang* sp. nov., holotype male (**C–F**) and paratype female (**A, B, G, H**). **A** Epigyne, ventral view **B** vulva, dorsal view **C** bulbal apophyses, prolateral view, arrow points at “pseudo-appendix” **D** chelicerae, frontal view **E–H** habitus (**E, G** dorsal view **F** lateral view **H** ventral view). Abbreviations: aa = anterior arch, b = bulb, da = distal apophysis, e = embolus, fa = frontal apophysis, kn = knob, pa = proximo-lateral apophysis, pp = pore plate, u = uncus. Scale bars: 0.20 mm (**A–D**); 1.00 mm (**E–H**).

The new species resembles *Pholcushuailai* Yao, Li & Lu, 2022 (Lu et al. 2022: 534, S18, figs S17A–D, S18A–H) by having a similar uncus (Fig. 4C) and male chelicerae (Fig. 4D), but it can be distinguished by the: procursus with a raised, prolatero-subdistal membranous edge (arrow 1 in Fig. 3C vs. absent), the straight distal edge of the sclerotized distal apophysis on the procursus (arrow 1 in Fig. 3D vs. curved), epigynal knob column shaped (kn in Fig. 4A vs. wedge shaped), vulval anterior arch curved (aa in Fig. 4B vs. nearly trapezoidal).

##### Description.

**Male** (***holotype***): Total length 4.95 (5.14 with clypeus), carapace 1.55 long, 1.88 wide, opisthosoma 3.40 long, 1.60 wide. Leg I: 36.07 (9.29, 0.76, 9.10, 14.62, 2.30), leg II: 25.27 (7.12, 0.72, 6.35, 9.55, 1.53), leg III: 18.79 (5.45, 0.67, 4.55, 6.99, 1.13), leg IV: 24.69 (7.24, 0.68, 6.22, 9.10, 1.45); tibia I L/d: 55. Eye interdistances and diameters: PME–PME 0.21, PME 0.14, PME–ALE 0.04, AME–AME 0.04, AME 0.09. Sternum width/length: 1.19/0.98. Habitus as in Fig. 4E, F. Carapace yellowish, with brown radiating marks and marginal brown bands; ocular area yellowish, with median and lateral brown bands; clypeus yellowish, with brown marks; sternum brown. Legs yellowish, dark brown on patellae and whitish on distal parts of femora and tibiae, with darker rings on subdistal parts of femora and proximal and subdistal parts of tibiae. Opisthosoma yellowish, with dorsal and lateral brown spots. Chelicerae with pair of proximo-lateral apophyses (pa in Fig. 4D), pair of distal apophyses (da in Fig. 4D) with two teeth each, and pair of frontal apophyses (fa in Fig. 4D). Palps as in Fig. 3A, B; trochanter with long (3 × longer than wide), retrolaterally bulged ventral apophysis; femur with retrolatero-proximal protrusion (arrow in Fig. 3B) and indistinct ventral protrusion; tibia with prolatero-ventral protrusion (arrow in Fig. 3A); procursus simple proximally but complex distally, with raised, prolatero-subdistal membranous edge bearing sclerotized apophysis (arrows 1, 2 in Fig. 3C), sclerotized distal apophysis (arrow 3 in Fig. 3C), and one slender and one strong dorsal spines (arrows 2, 3 in Fig. 3D); uncus with scaly edge (u in Fig. 4C); “pseudo-appendix” curved (arrow in Fig. 4C); embolus weakly sclerotized, with transparent distal projections (e in Fig. 4C). Retrolateral trichobothrium on tibia I at 6% proximally; legs with short, vertical setae on tibiae, metatarsi, and tarsi; tarsus I with 31 distinct pseudosegments.

**Female** (***paratype***, SYNU-Ar00417): Similar to male, habitus as in Fig. 4G, H. Total length 5.12 (5.26 with clypeus), carapace 1.44 long, 1.66 wide, opisthosoma 3.68 long, 2.08 wide; tibia I: 7.18; tibia I L/d: 48. Eye interdistances and diameters: PME–PME 0.23, PME 0.12, PME–ALE 0.05, AME–AME 0.04, AME 0.08. Sternum width/length: 1.05/0.94. Clypeus brown. Epigyne posteriorly strongly curved, with lateral brown marks and knob (kn in Fig. 4A). Vulva with curved, sclerotized anterior arch (aa in Fig. 4B) and pair of nearly elliptical pore plates (pp in Fig. 4B).

##### Variation.

Tibia I in paratype male (SYNU-Ar00416): 9.23. Tibia I in another paratype female (SYNU-Ar00418): 7.24.

##### Habitat.

Underside of overhang on rocky cliffs in mountainous area.

##### Distribution.

Liaoning; known only from the type locality (Figs 1, 2A).

#### ﻿ Pholcus hebei

Taxon classificationAnimaliaAraneaePholcidae

S. Li & Yao sp. nov.

B26D19C2-B620-5CDB-9EA2-8BBC4932ADDC

https://zoobank.org/094C644B-DC6B-4776-983B-767069BD3DFD

Figs 5 , 6

##### Type material.

***Holotype***: China • ♂; Hebei, Chengde, Longhua County, Zhongguan Town, Longfengdong Scenic Spot; 41.186398°N, 117.988977°E; alt. 496 m; 19 Jul. 2024; Z. Yao, J. Li & M. Yan leg.; SYNU-Ar00419. ***Paratypes***: China • 1♂; same data as for the holotype; SYNU-Ar00420 • 2♀; same data as for the holotype; SYNU-Ar00421–22.

##### Etymology.

The specific name refers to the type locality; noun in apposition.

##### Diagnosis.

**Figure 5.**
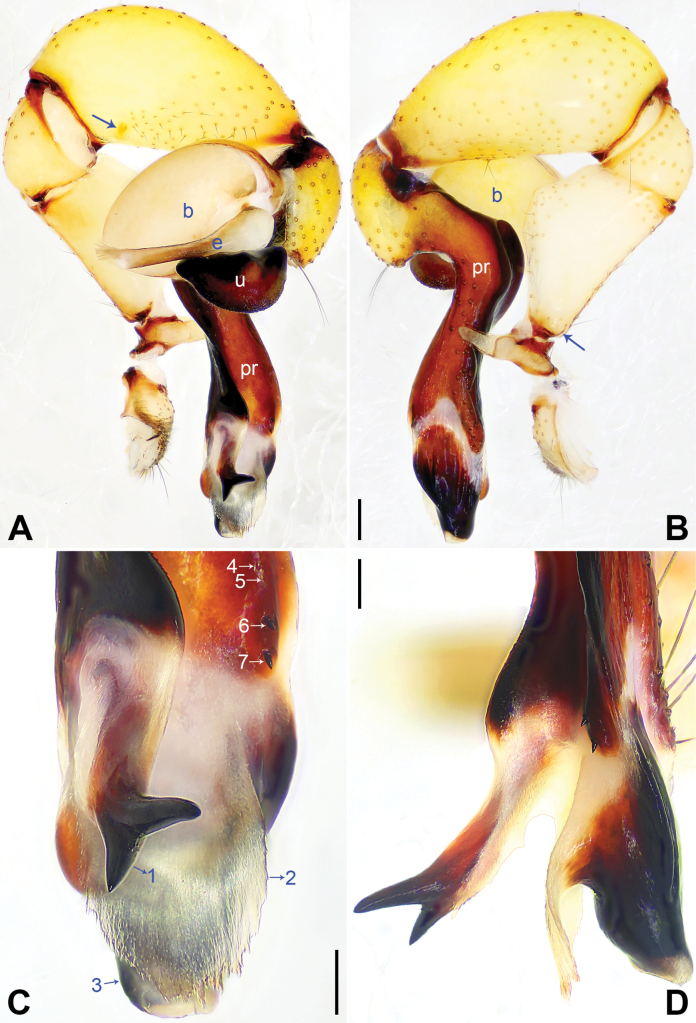
*Pholcushebei* sp. nov., holotype male. **A, B** Palp (**A** prolateral view, arrow points at prolatero-ventral protrusion **B** retrolateral view, arrow points at retrolatero-proximal protrusion) **C, D** distal part of procursus (**C** prolateral view, arrow 1 points at bifurcated apophysis, arrow 2 points at distal membranous lamella, arrow 3 points at distal apophysis, arrows 4–7 point at dorsal spines **D** dorsal view). Abbreviations: b = bulb, e = embolus, pr = procursus, u = uncus. Scale bars: 0.20 mm (**A, B**); 0.10 mm (**C, D**).

**Figure 6.**
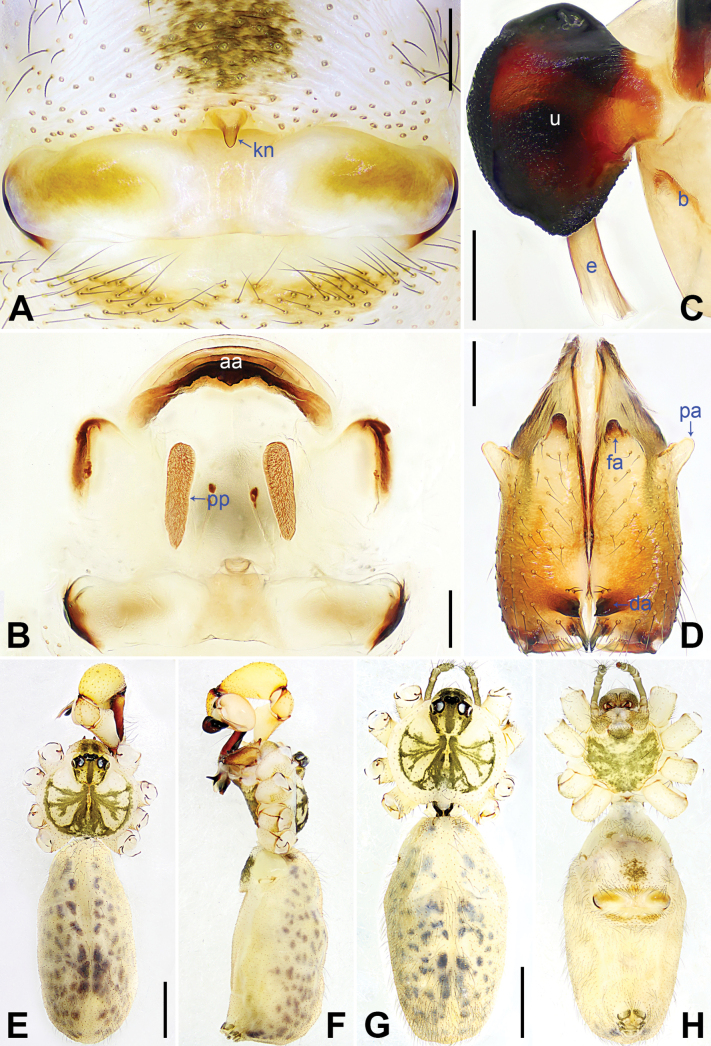
*Pholcushebei* sp. nov., holotype (**D–F**) and paratype (**C**) males, paratype female (**A, B, G, H**). **A** Epigyne, ventral view **B** vulva, dorsal view **C** bulbal apophyses, prolateral view **D** chelicerae, frontal view **E–H** habitus (**E, G** dorsal view **F** lateral view **H** ventral view). Abbreviations: aa = anterior arch, b = bulb, da = distal apophysis, e = embolus, fa = frontal apophysis, kn = knob, pa = proximo-lateral apophysis, pp = pore plate, u = uncus. Scale bars: 0.20 mm (**A–D**); 1.00 mm (**E–H**).

The new species resembles *Pholcuszhuolu* Zhang & Zhu, 2009 (Zhang and Zhu 2009: 108, figs 64A–I, 65A–L, Yao and Li 2012: 43, figs 225A–D, 226A–C) by having a similar uncus (Fig. 6C) and male chelicerae (Fig. 6D), but it can be distinguished by the: procursus with a bifurcated prolatero-subdistal apophysis (arrow 1 in Fig. 5C vs. not bifurcated), the large distal membranous lamella (arrow 2 in Fig. 5C vs. indistinct), epigynal plate anteriorly straight (Fig. 6A vs. strongly curved), vulval pore plates long, 4 × longer than wide (pp in Fig. 6B vs. 2 ×).

##### Description.

**Male** (***holotype***): Total length 5.37 (5.67 with clypeus), carapace 1.52 long, 1.83 wide, opisthosoma 3.85 long, 1.80 wide. Leg I: 43.25 (10.96, 0.75, 11.03, 17.88, 2.63), leg II: 29.18 (8.01, 0.72, 7.31, 11.41, 1.73), leg III: 20.24 (5.83, 0.69, 4.81, 7.63, 1.28), leg IV: 26.48 (7.56, 0.71, 6.41, 10.26, 1.54); tibia I L/d: 74. Eye interdistances and diameters: PME–PME 0.24, PME 0.16, PME–ALE 0.04, AME–AME 0.05, AME 0.09. Sternum width/length: 1.25/1.03. Habitus as in Fig. 6E, F. Carapace yellowish, with brown radiating marks and marginal brown bands; ocular area yellowish, with median and lateral brown bands; clypeus and sternum yellowish, with brown marks. Legs yellowish, dark brown on patellae and whitish on distal parts of femora and tibiae, with darker rings on subdistal parts of femora and proximal and subdistal parts of tibiae. Opisthosoma yellowish, with dorsal and lateral brown spots. Chelicerae with pair of proximo-lateral apophyses (pa in Fig. 6D), pair of distal apophyses (da in Fig. 6D) with two teeth each, and pair of frontal apophyses (fa in Fig. 6D). Palps as in Fig. 5A, B; trochanter with long (4 × longer than wide), retrolaterally strongly bulged ventral apophysis; femur with retrolatero-proximal protrusion (arrow in Fig. 5B) and indistinct ventral protrusion; tibia with prolatero-ventral protrusion (arrow in Fig. 5A); procursus simple proximally but complex distally, with raised prolatero-subdistal edge bearing bifurcated apophysis (arrow 1 in Fig. 5C), distal membranous lamella (arrow 2 in Fig. 5C), distal apophysis (arrow 3 in Fig. 5C), and one slender and three strong dorsal spines (arrows 4–7 in Fig. 5C); uncus nearly elliptical, with scales (u in Fig. 6C); “pseudo-appendix” semi-transparent (not visible in Fig. 6C; cf. Fig. 8C); embolus weakly sclerotized, with transparent distal projections (e in Fig. 6C). Retrolateral trichobothrium on tibia I at 5% proximally; legs with short, vertical setae on tibiae, metatarsi, and tarsi; tarsus I with 36 distinct pseudosegments.

**Female** (***paratype***, SYNU-Ar00421): Similar to male, habitus as in Fig. 6G, H. Total length 4.83 (5.01 with clypeus), carapace 1.48 long, 1.70 wide, opisthosoma 3.35 long, 1.84 wide; tibia I: 8.01; tibia I L/d: 53. Eye interdistances and diameters: PME–PME 0.20, PME 0.13, PME–ALE 0.05, AME–AME 0.04, AME 0.08. Sternum width/length: 1.16/0.91. Clypeus brown. Epigyne posteriorly straight, with lateral and median brown marks and knob (kn in Fig. 6A). Vulva with curved, sclerotized anterior arch (aa in Fig. 6B) and pair of long, elliptical pore plates (4 × longer than wide, pp in Fig. 6B).

##### Variation.

Tibia I in paratype male (SYNU-Ar00420): 11.54. Tibia I in another paratype female (SYNU-Ar00422): 7.56.

##### Habitat.

Underside of overhang on rocky cliffs in mountainous area.

##### Distribution.

Hebei; known only from the type locality (Figs 1, 2B).

#### ﻿ Pholcus huludao

Taxon classificationAnimaliaAraneaePholcidae

S. Li & Yao sp. nov.

34B99749-4B01-57BA-AFF6-E97242016F16

https://zoobank.org/A1DDEF77-6E27-4935-B248-22B7819E8711

Figs 7 , 8

##### Type material.

***Holotype***: China • ♂; Liaoning, Huludao, Jianchang County, Laodazhangzi Town, Longtan Grand Canyon Scenic Spot; 40.554405°N, 119.349993°E; alt. 504 m; 21 Jul. 2024; Z. Yao, J. Li & M. Yan leg.; SYNU-Ar00423. ***Paratypes***: China • 1♂; same data as for the holotype; SYNU-Ar00424 • 2♀; same data as for the holotype; SYNU-Ar00425–26.

##### Etymology.

The specific name refers to the type locality; noun in apposition.

##### Diagnosis.

**Figure 7.**
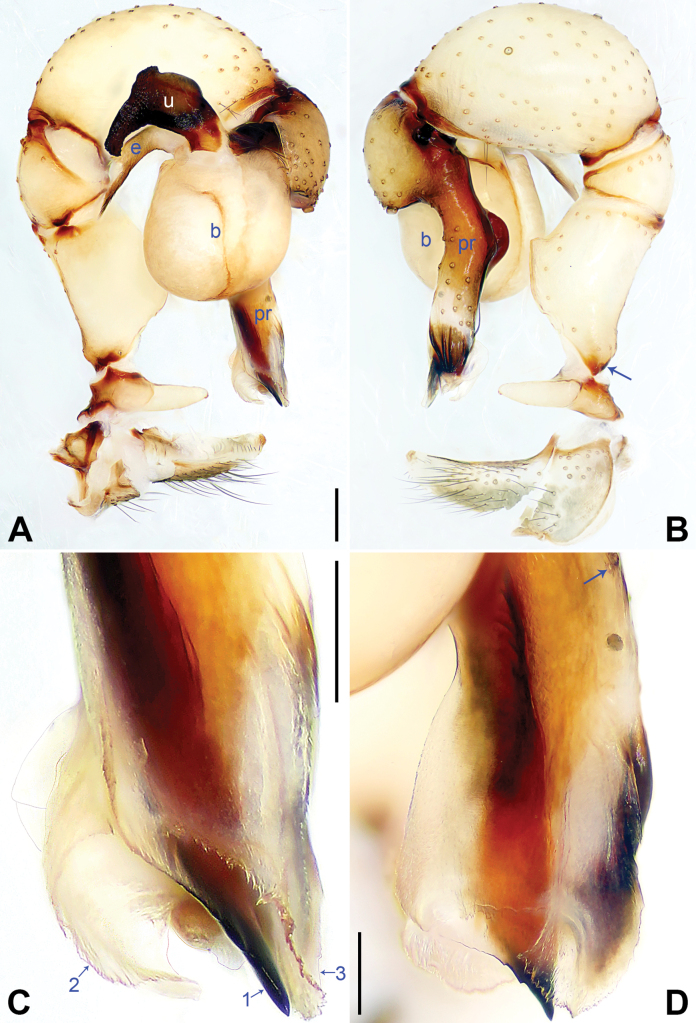
*Pholcushuludao* sp. nov., holotype male. **A, B** Palp (**A** prolateral view **B** retrolateral view, arrow points at retrolatero-proximal protrusion) **C, D** distal part of procursus (**C** prolateral view, arrow 1 points at pointed sclerotized apophysis, arrow 2 points at ventro-distal membranous lamella, arrow 3 points at dorso-distal membranous lamella **D** dorsal view, arrow points at dorsal spine). Abbreviations: b = bulb, e = embolus, pr = procursus, u = uncus. Scale bars: 0.20 mm (**A, B**); 0.10 mm (**C, D**).

**Figure 8.**
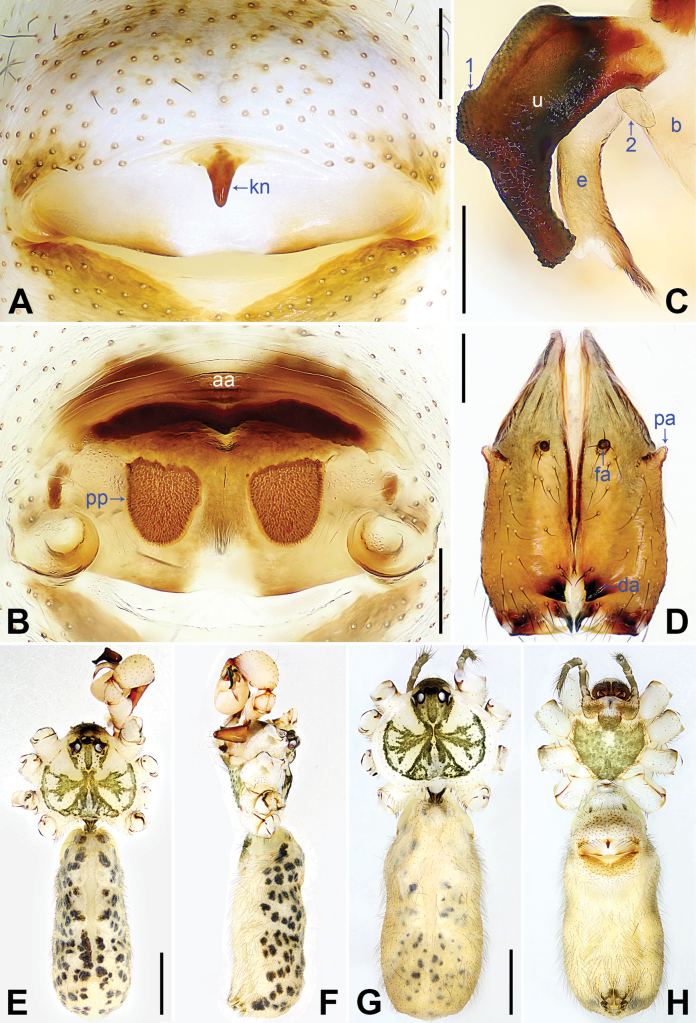
*Pholcushuludao* sp. nov., holotype male (**C–F**) and paratype female (**A, B, G, H**). **A** Epigyne, ventral view **B** vulva, dorsal view **C** bulbal apophyses, prolateral view, arrow 1 points at dorso-median protrusion, arrow 2 points at “pseudo-appendix” **D** chelicerae, frontal view **E–H** habitus (**E, G** dorsal view **F** lateral view **H** ventral view). Abbreviations: aa = anterior arch, b = bulb, da = distal apophysis, e = embolus, fa = frontal apophysis, kn = knob, pa = proximo-lateral apophysis, pp = pore plate, u = uncus. Scale bars: 0.20 mm (**A–D**); 1.00 mm (**E–H**).

The new species resembles known congeners from the Lüliang Mountains (e.g., *Pholcuswenshui* Zhao, Li & Yao, 2023, *P.jiaocheng* Zhao, Li & Yao, 2023, *P.luliang* Zhao, Li & Yao, 2023, *P.zhongyang* Zhao, Li & Yao, 2023; Zhao et al. 2023b: 7, figs 2B, 4C, D, 10C, D, 12C, D, 18C, D) by having similar male chelicerae (Fig. 8D) and a curved uncus (Fig. 8C), but it can be distinguished by the following combination of characters: ventro-distal membranous lamella of procursus distally blunt and strongly curved (arrow 2 in Fig. 7C vs. distally pointed and straight), dorso-distal membranous lamella of procursus strongly curved (arrow 3 in Fig. 7C vs. straight), uncus proximally wide and distally narrow, and dorso-medially strongly protruding (arrow 1 in Fig. 8C vs. wide and pointed, not protruding), epigyne posteriorly slightly curved (Fig. 8A vs. strongly curved), vulval pore plates nearly semi-circular (pp in Fig. 8B vs. elliptical).

##### Description.

**Male** (***holotype***): Total length 4.68 (4.80 with clypeus), carapace 1.44 long, 1.72 wide, opisthosoma 3.24 long, 1.27 wide. Leg I: 39.99 (9.94, 0.75, 10.13, 16.67, 2.50), leg II: 27.14 (7.45, 0.67, 6.73, 10.64, 1.65), leg III: 18.98 (5.58, 0.64, 4.62, 7.05, 1.09), leg IV: 25.51 (7.40, 0.66, 6.35, 9.62, 1.48); tibia I L/d: 68. Eye interdistances and diameters: PME–PME 0.23, PME 0.15, PME–ALE 0.04, AME–AME 0.05, AME 0.10. Sternum width/length: 1.17/0.98. Habitus as in Fig. 8E, F. Carapace yellowish, with brown radiating marks and marginal brown bands; ocular area yellowish, with median and lateral brown bands; clypeus brown; sternum yellowish, with brown marks. Legs yellowish, dark brown on patellae and whitish on distal parts of femora and tibiae, with darker rings on subdistal parts of femora and proximal and subdistal parts of tibiae. Opisthosoma yellowish, with dorsal and lateral brown spots. Chelicerae with pair of proximo-lateral apophyses (pa in Fig. 8D), pair of distal apophyses (da in Fig. 8D) with two teeth each, and pair of frontal apophyses (fa in Fig. 8D). Palps as in Fig. 7A, B; trochanter with long (4 × longer than wide), retrolaterally bulged ventral apophysis; femur with retrolatero-proximal protrusion (arrow in Fig. 7B) and distinct ventral protrusion; tibia with prolatero-ventral protrusion; procursus simple proximally but complex distally, with raised, prolatero-subdistal membranous edge bearing pointed, sclerotized apophysis (arrow 1 in Fig. 7C), curved, ventro-distal membranous lamella (arrow 2 in Fig. 7C), curved, dorso-distal membranous lamella (arrow 3 in Fig. 7C), and one slender dorsal spine (arrow in Fig. 7D); uncus curved, proximally wide and distally narrow, with scales (u in Fig. 8C); “pseudo-appendix” semi-transparent (arrow 2 in Fig. 8C); embolus weakly sclerotized, with transparent distal projections (e in Fig. 8C). Retrolateral trichobothrium on tibia I at 2% proximally; legs with short, vertical setae on tibiae, metatarsi, and tarsi; tarsus I with 30 distinct pseudosegments.

**Female** (***paratype***, SYNU-Ar00425): Similar to male, habitus as in Fig. 8G, H. Total length 5.13 (5.26 with clypeus), carapace 1.53 long, 1.95 wide, opisthosoma 3.60 long, 1.60 wide; tibia I: 8.78; tibia I L/d: 59. Eye interdistances and diameters: PME–PME 0.21, PME 0.14, PME–ALE 0.05, AME–AME 0.05, AME 0.10. Sternum width/length: 1.11/0.97. Epigyne posteriorly slightly curved, with lateral brown marks and knob (kn in Fig. 8A). Vulva with curved, sclerotized anterior arch (aa in Fig. 8B) and pair of nearly semi-circular pore plates (pp in Fig. 8B).

##### Variation.

Tibia I in paratype male (SYNU-Ar00424): 9.80. Tibia I in another paratype female (SYNU-Ar00426): 9.25.

##### Habitat.

Underside of overhang on rocky cliffs in mountainous area.

##### Distribution.

Liaoning; known only from the type locality (Figs 1, 2C).

#### ﻿ Pholcus jinzhou

Taxon classificationAnimaliaAraneaePholcidae

S. Li & Yao sp. nov.

62C243BB-7FC1-511E-BD1A-D79E8BAB2CC1

https://zoobank.org/8C1DC153-00D3-4BDF-8301-F675291E3479

Figs 9 , 10

##### Type material.

***Holotype***: China • ♂; Liaoning, Jinzhou, Guta District, Beiputuoshan Scenic Spot; 41.173150°N, 121.042606°E; alt. 163 m; 15 Jul. 2024; Z. Yao, J. Li & M. Yan leg.; SYNU-Ar00427. ***Paratypes***: China • 2♂; same data as for the holotype; SYNU-Ar00428–29 • 3♀; same data as for the holotype; SYNU-Ar00430–32.

##### Etymology.

The specific name refers to the type locality; noun in apposition.

##### Diagnosis.

**Figure 9.**
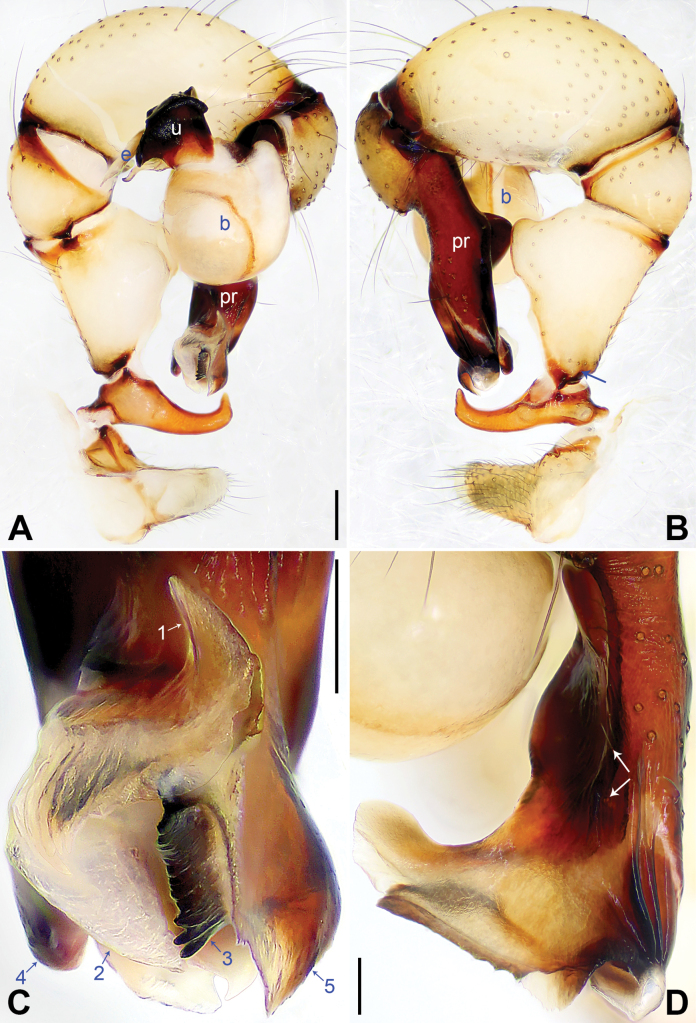
*Pholcusjinzhou* sp. nov., holotype male. **A, B** Palp (**A** prolateral view **B** retrolateral view, arrow points at retrolatero-proximal protrusion) **C, D** distal part of procursus (**C** prolateral view, arrow 1 points at curved apophysis, arrow 2 points at membranous process, arrow 3 points at comb-like sclerotized apophysis, arrow 4 points at ventro-distal apophysis, arrow 5 points at dorso-distal apophysis **D** dorsal view, arrows point at dorsal spines). Abbreviations: b = bulb, e = embolus, pr = procursus, u = uncus. Scale bars: 0.20 mm (**A, B**); 0.10 mm (**C, D**).

**Figure 10.**
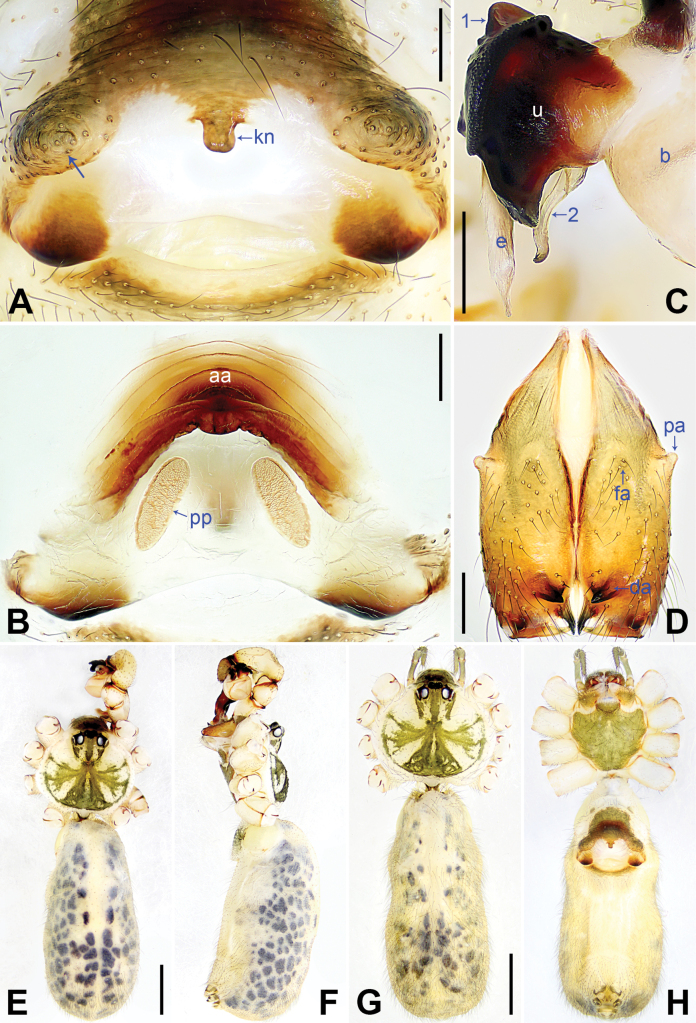
*Pholcusjinzhou* sp. nov., holotype male (**C–F**) and paratype female (**A, B, G, H**). **A** Epigyne, ventral view, arrow points at lateral protrusion **B** vulva, dorsal view **C** bulbal apophyses, prolateral view, arrow 1 points at retrolatero-proximal apophysis, arrow 2 points at “pseudo-appendix” **D** chelicerae, frontal view **E–H** habitus (**E, G** dorsal view **F** lateral view **H** ventral view). Abbreviations: aa = anterior arch, b = bulb, da = distal apophysis, e = embolus, fa = frontal apophysis, kn = knob, pa = proximo-lateral apophysis, pp = pore plate, u = uncus. Scale bars: 0.20 mm (**A–D**); 1.00 mm (**E–H**).

The new species can be easily distinguished from all known congeners in the *Pholcusphungiformes* species group by the following combination of characters: procursus with curved prolatero-subdistal apophysis (arrow 1 in Fig. 9C vs. absent) and comb-like, sclerotized prolatero-distal apophysis (arrow 3 in Fig. 9C vs. absent), proximal apophysis of uncus on retrolateral part of uncus (arrow 1 in Fig. 10C vs. same plane or absent), epigyne with pair of lateral protrusions anterior to epigynal plate (arrow in Fig. 10A vs. absent).

##### Description.

**Male** (***holotype***): Total length 5.78 (6.01 with clypeus), carapace 1.73 long, 1.95 wide, opisthosoma 4.05 long, 1.93 wide. Leg I: 43.34 (11.09, 0.78, 11.54, 17.56, 2.37), leg II: 30.61 (8.27, 0.83, 7.82, 12.05, 1.64), leg III: 21.87 (6.35, 0.73, 5.32, 8.21, 1.26), leg IV: 28.41 (8.08, 0.73, 7.24, 10.77, 1.59); tibia I L/d: 68. Eye interdistances and diameters: PME–PME 0.28, PME 0.16, PME–ALE 0.03, AME–AME 0.05, AME 0.11. Sternum width/length: 1.30/0.94. Habitus as in Fig. 10E, F. Carapace brown, with brown radiating marks and marginal brown bands; ocular area yellowish, with median and lateral brown bands; clypeus and sternum brown. Legs yellowish, dark brown on patellae and whitish on distal parts of femora and tibiae, with darker rings on subdistal parts of femora and proximal and subdistal parts of tibiae. Opisthosoma yellowish, with dorsal and lateral brown spots. Chelicerae with pair of proximo-lateral apophyses (pa in Fig. 10D), pair of distal apophyses (da in Fig. 10D) with two teeth each, and pair of frontal apophyses (fa in Fig. 10D). Palps as in Fig. 9A, B; trochanter with long (6 × longer than wide), retrolaterally strongly bulged ventral apophysis; femur with retrolatero-proximal protrusion (arrow in Fig. 9B) and distinct ventral protrusion; tibia with prolatero-ventral protrusion; procursus simple proximally but complex distally, with raised prolatero-subdistal edge bearing curved apophysis (arrow 1 in Fig. 9C), membranous process (arrow 2 in Fig. 9C) and comb-like sclerotized apophysis (arrow 3 in Fig. 9C), ventro-distal apophysis (arrow 4 in Fig. 9C), dorso-distal apophysis (arrow 5 in Fig. 9C), and two slender dorsal spines (arrows in Fig. 9D); uncus with distinct retrolatero-proximal apophysis (arrow 1 in Fig. 10C) and scaly edge; “pseudo-appendix” curved, distally sclerotized (arrow 2 in Fig. 10C); embolus weakly sclerotized, with transparent distal projections (e in Fig. 10C). Retrolateral trichobothrium on tibia I at 3% proximally; legs with short, vertical setae on tibiae, metatarsi, and tarsi; tarsus I with 35 distinct pseudosegments.

**Female** (***paratype***, SYNU-Ar00430): Similar to male, habitus as in Fig. 10G, H. Total length 5.56 (5.80 with clypeus), carapace 1.66 long, 2.05 wide, opisthosoma 3.90 long, 1.70 wide; tibia I: 9.94; tibia I L/d: 55. Eye interdistances and diameters: PME–PME 0.25, PME 0.15, PME–ALE 0.05, AME–AME 0.05, AME 0.09. Sternum width/length: 1.24/1.12. Epigyne posteriorly curved, with lateral brown marks, knob (kn in Fig. 10A), and pair of lateral protrusions anterior to epigynal plate (arrow in Fig. 10A). Vulva with curved, sclerotized anterior arch (aa in Fig. 10B) and pair of long, elliptical pore plates (3 × longer than wide, pp in Fig. 10B).

##### Variation.

Tibia I in two paratype males (SYNU-Ar00428–29): 11.15, 11.54. Tibia I in the other two paratype females (SYNU-Ar00431–32): 9.36, 9.42.

##### Habitat.

Underside of overhang on rocky cliffs in mountainous area.

##### Distribution.

Liaoning; known only from the type locality (Figs 1, 2D).

#### ﻿ Pholcus liaoning

Taxon classificationAnimaliaAraneaePholcidae

S. Li & Yao sp. nov.

7A4D2C6B-4A51-5939-A859-C150CAA25F6B

https://zoobank.org/D0822275-FCA9-404D-81C2-D7C108277EB6

Figs 11 , 12

##### Type material.

***Holotype***: China • ♂; Liaoning, Chaoyang, Beipiao, Daheishan Scenic Spot; 42.006997°N, 120.484051°E; alt. 609 m; 16 Jul. 2024; Z. Yao, J. Li & M. Yan leg.; SYNU-Ar00433. ***Paratypes***: China • 1♂; same data as for the holotype; SYNU-Ar00434 • 2♀; same data as for the holotype; SYNU-Ar00435–36.

##### Etymology.

The specific name refers to the type locality; noun in apposition.

##### Diagnosis.

**Figure 11.**
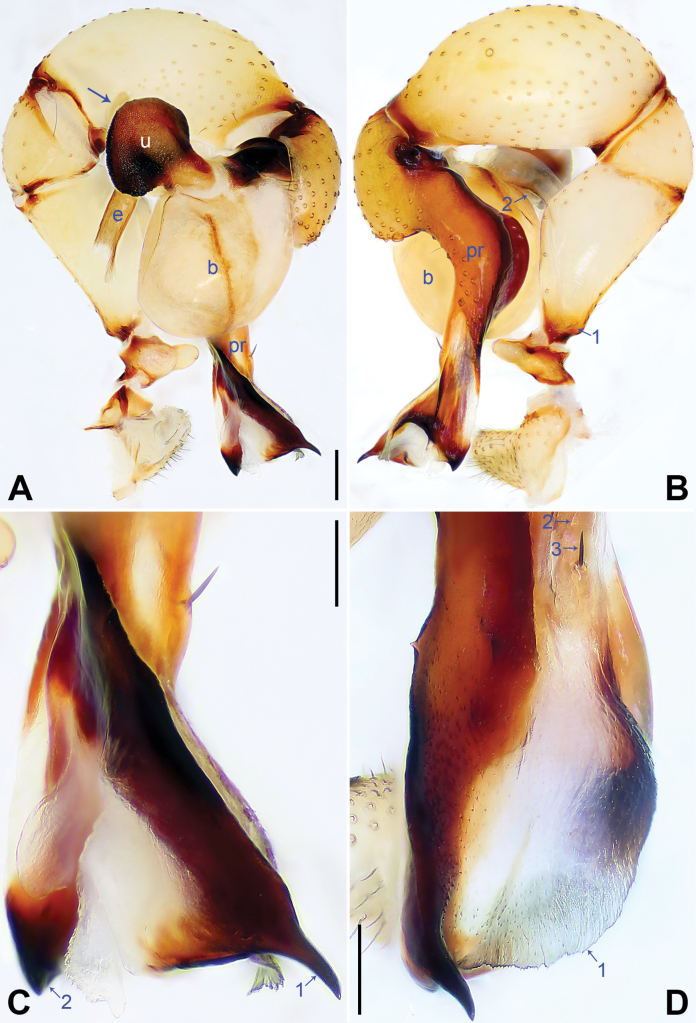
*Pholcusliaoning* sp. nov., holotype male. **A, B** Palp (**A** prolateral view, arrow points at prolatero-ventral protrusion **B** retrolateral view, arrow 1 points at retrolatero-proximal protrusion, arrow 2 points at “pseudo-appendix”) **C, D** distal part of procursus (**C** prolateral view, arrow 1 points at pointed apophysis, arrow 2 points at ventro-distal apophysis **D** dorsal view, arrow 1 points at dorso-distal membranous lamella, arrows 2, 3 point at dorsal spines). Abbreviations: b = bulb, e = embolus, pr = procursus, u = uncus. Scale bars: 0.20 mm (**A, B**); 0.10 mm (**C, D**).

**Figure 12.**
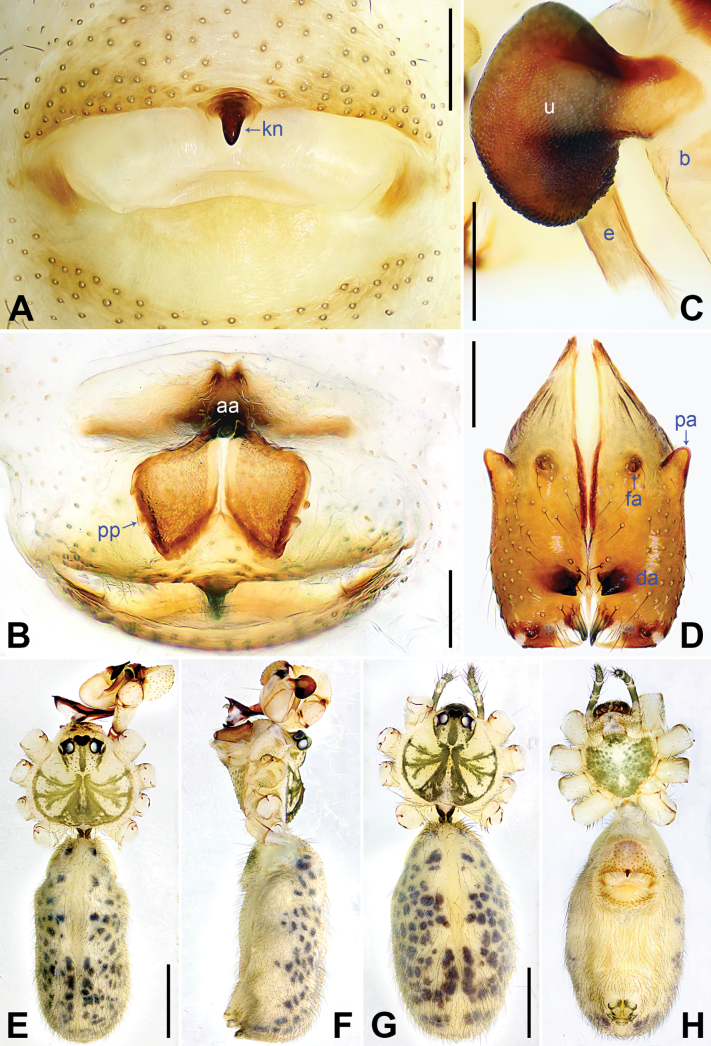
*Pholcusliaoning* sp. nov., holotype male (**C–F**) and paratype female (**A, B, G, H**). **A** Epigyne, ventral view **B** vulva, dorsal view **C** bulbal apophyses, prolateral view **D** chelicerae, frontal view **E–H** habitus (**E, G** dorsal view **F** lateral view **H** ventral view). Abbreviations: aa = anterior arch, b = bulb, da = distal apophysis, e = embolus, fa = frontal apophysis, kn = knob, pa = proximo-lateral apophysis, pp = pore plate, u = uncus. Scale bars: 0.20 mm (**A–D**); 1.00 mm (**E–H**).

The new species resembles *Pholcuswangjiang* Yao & Li, 2021 (Yao et al. 2021: S22, figs 2B.20, S21A–D, S22A–H) by having a similar uncus (Fig. 12C) and male chelicerae (Fig. 12D), but it can be distinguished by the: procursus with a ventro-distal apophysis (arrow 2 in Fig. 11C vs. absent) and nearly semi-circular, dorso-distal membranous lamella (arrow 1 in Fig. 11D vs. absent), epigynal plate without brown marks (Fig. 12A vs. with distinct lateral brown marks), vulval pore plates quadrilateral (pp in Fig. 12B vs. elliptical).

##### Description.

**Male** (***holotype***): Total length 4.37 (4.49 with clypeus), carapace 1.34 long, 1.50 wide, opisthosoma 3.03 long, 1.41 wide. Leg I: 37.15 (9.36, 0.70, 9.49, 15.32, 2.28), leg II: 25.89 (7.12, 0.67, 6.47, 10.13, 1.50), leg III: 18.46 (5.38, 0.63, 4.49, 6.79, 1.17), leg IV: 24.22 (6.99, 0.63, 6.20, 9.04, 1.36); tibia I L/d: 67. Eye interdistances and diameters: PME–PME 0.21, PME 0.14, PME–ALE 0.04, AME–AME 0.06, AME 0.08. Sternum width/length: 1.04/0.86. Habitus as in Fig. 12E, F. Carapace yellowish, with brown radiating marks and marginal brown bands; ocular area yellowish, with median and lateral brown bands; clypeus and sternum yellowish, with brown marks. Legs yellowish, dark brown on patellae and whitish on distal parts of femora and tibiae, with darker rings on subdistal parts of femora and proximal and subdistal parts of tibiae. Opisthosoma yellowish, with dorsal and lateral brown spots. Chelicerae with pair of proximo-lateral apophyses (pa in Fig. 12D), pair of distal apophyses (da in Fig. 12D) with two teeth each, and pair of frontal apophyses (fa in Fig. 12D). Palps as in Fig. 11A, B; trochanter with retrolaterally strongly bulged ventral apophysis; femur with retrolatero-proximal protrusion (arrow 1 in Fig. 11B) and indistinct ventral protrusion; tibia with prolatero-ventral protrusion (arrow in Fig. 11A); procursus simple proximally but complex distally, with raised prolatero-subdistal edge bearing pointed apophysis (arrow 1 in Fig. 11C), ventro-distal apophysis (arrow 2 in Fig. 11C), dorso-distal membranous lamella (arrow 1 in Fig. 11D), and two strong dorsal spines (arrows 2, 3 in Fig. 11D); uncus nearly elliptical, with scales (u in Fig. 12C); “pseudo-appendix” semi-transparent (arrow 2 in Fig. 11B); embolus weakly sclerotized, with transparent distal projections (e in Fig. 12C). Retrolateral trichobothrium on tibia I at 5% proximally; legs with short, vertical setae on tibiae, metatarsi, and tarsi; tarsus I with 30 distinct pseudosegments.

**Female** (***paratype***, SYNU-Ar00435): Similar to male, habitus as in Fig. 12G, H. Total length 4.68 (4.81 with clypeus), carapace 1.40 long, 1.52 wide, opisthosoma 3.28 long, 1.86 wide; tibia I: 8.01; tibia I L/d: 57. Eye interdistances and diameters: PME–PME 0.19, PME 0.13, PME–ALE 0.04, AME–AME 0.04, AME 0.08. Sternum width/length: 0.99/0.90. Clypeus brown. Epigyne posteriorly slightly curved, with knob (kn in Fig. 12A). Vulva with ridge-shaped anterior arch (aa in Fig. 12B) and pair of quadrilateral pore plates (pp in Fig. 12B).

##### Variation.

Tibia I in paratype male (SYNU-Ar00434): 8.84. Tibia I in another paratype female (SYNU-Ar00436): 8.33.

##### Habitat.

Underside of overhang on rocky cliffs in mountainous area.

##### Distribution.

Liaoning; known only from the type locality (Figs 1, 2E).

#### ﻿ Pholcus qin

Taxon classificationAnimaliaAraneaePholcidae

S. Li & Yao sp. nov.

4CA994D4-0645-5C9B-A564-82DDC08F1377

https://zoobank.org/49F606E5-B2C4-4265-8757-3377DA59AB49

Figs 13 , 14

##### Type material.

***Holotype***: China • ♂; Hebei, Qinhuangdao, Funing District, Daxinzhai Town, Bingtangyu Scenic Spot; 40.106112°N, 119.356216°E; alt. 250 m; 20 Jul. 2024; Z. Yao, J. Li & M. Yan leg.; SYNU-Ar00437. ***Paratypes***: China • 2♂; same data as for the holotype; SYNU-Ar00438–39 • 3♀; same data as for the holotype; SYNU-Ar00440–42.

##### Etymology.

The specific name refers to the type locality (Qin is a short name for Qinhuangdao); noun in apposition.

##### Diagnosis.

**Figure 13.**
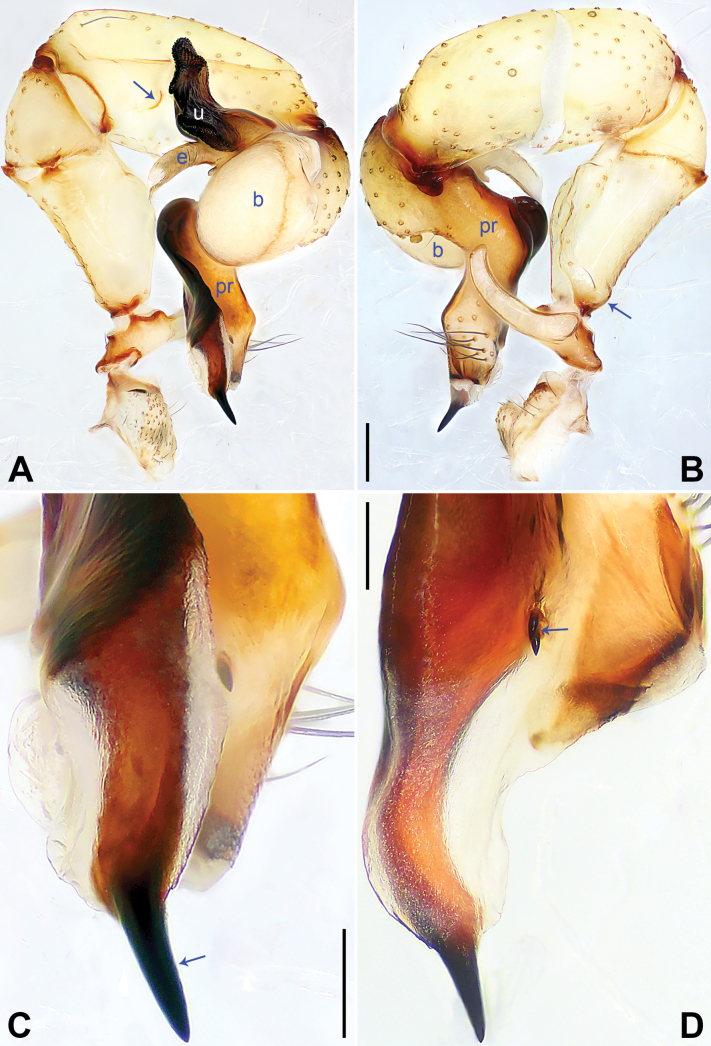
*Pholcusqin* sp. nov., holotype male. **A, B** Palp (**A** prolateral view, arrow points at prolatero-ventral protrusion **B** retrolateral view, arrow points at retrolatero-proximal protrusion) **C, D** distal part of procursus (**C** prolateral view, arrow points at spine-shaped apophysis **D** dorsal view, arrow points at dorsal spines). Abbreviations: b = bulb, e = embolus, pr = procursus, u = uncus. Scale bars: 0.20 mm (**A, B**); 0.10 mm (**C, D**).

**Figure 14.**
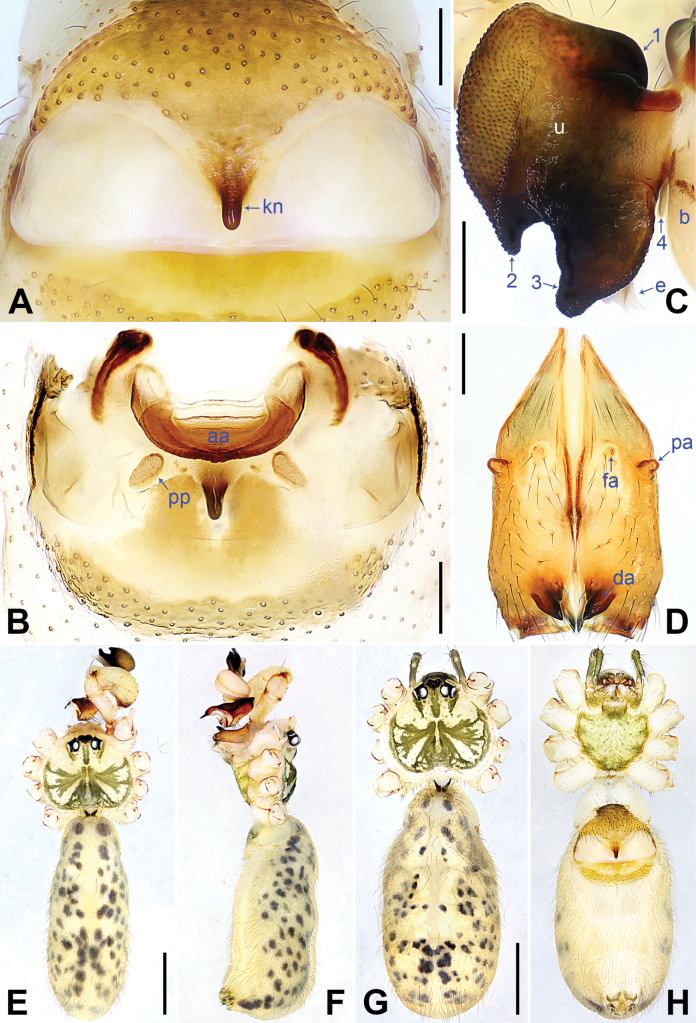
*Pholcusqin* sp. nov., holotype male (**C–F**) and paratype female (**A, B, G, H**). **A** Epigyne, ventral view **B** vulva, dorsal view **C** bulbal apophyses, prolateral view, arrow 1 points at proximal apophysis, arrows 2, 3 point at distal apophyses, arrow 4 points at “pseudo-appendix” **D** chelicerae, frontal view **E–H** habitus (**E, G** dorsal view **F** lateral view **H** ventral view). Abbreviations: aa = anterior arch, b = bulb, da = distal apophysis, e = embolus, fa = frontal apophysis, kn = knob, pa = proximo-lateral apophysis, pp = pore plate, u = uncus. Scale bars: 0.20 mm (**A–D**); 1.00 mm (**E–H**).

The new species resembles *Pholcuskuaile* Yao, Li & Lu, 2022 (Lu et al. 2022: 534, S21, figs S22A–D, S23A–H) by having similar male chelicerae (Fig. 14D) and a vulval anterior arch (Fig. 14B), but it can be distinguished by the: procursus without an angular ventral sclerite (Fig. 13B vs. present) and without a distal membranous process (Fig. 13C vs. present), uncus with two distal apophyses (arrows 2, 3 in Fig. 14C vs. one), epigynal plate 3 × longer than wide (Fig. 14A vs. 8 ×), vulval pore plates widely separated and same size as knob (pp in Fig. 14B vs. close to each other and 8 × larger than knob).

##### Description.

**Male** (***holotype***): Total length 4.64 (4.80 with clypeus), carapace 1.28 long, 1.60 wide, opisthosoma 3.36 long, 1.42 wide. Leg I: 40.72 (10.06, 0.75, 10.00, 17.25, 2.66), leg II: 26.95 (7.31, 0.63, 6.47, 10.96, 1.58), leg III: 18.78 (5.32, 0.58, 4.50, 7.25, 1.13), leg IV: 24.34 (6.60, 0.59, 6.09, 9.68, 1.38); tibia I L/d: 77. Eye interdistances and diameters: PME–PME 0.20, PME 0.13, PME–ALE 0.04, AME–AME 0.05, AME 0.08. Sternum width/length: 1.05/0.93. Habitus as in Fig. 14E, F. Carapace yellowish, with brown radiating marks and marginal brown bands; ocular area yellowish, with median and lateral brown bands; clypeus brownish; sternum yellowish, with brown marks. Legs yellowish, dark brown on patellae and whitish on distal parts of femora and tibiae, with darker rings on subdistal parts of femora and proximal and subdistal parts of tibiae. Opisthosoma yellowish, with dorsal and lateral brown spots. Chelicerae with pair of proximo-lateral apophyses (pa in Fig. 14D), pair of distal apophyses (da in Fig. 14D) with two teeth each, and pair of frontal apophyses (fa in Fig. 14D). Palps as in Fig. 13A, B; trochanter with long (8 × longer than wide), retrolaterally strongly bulged ventral apophysis; femur with retrolatero-proximal protrusion (arrow in Fig. 13B) and distinct ventral protrusion; tibia with prolatero-ventral protrusion (arrow in Fig. 13A); procursus simple proximally but complex distally, with raised prolatero-subdistal edge bearing spine-shaped apophysis (arrow in Fig. 13C), and two strong dorsal spines (arrow in Fig. 13D); uncus with scales, proximal apophysis (arrow 1 in Fig. 14C), and two distal apophyses (arrows 2, 3 in Fig. 14C); “pseudo-appendix” semi-transparent (arrow 4 in Fig. 14C); embolus weakly sclerotized, with transparent distal projections (e in Fig. 14C). Retrolateral trichobothrium on tibia I at 3% proximally; legs with short, vertical setae on tibiae, metatarsi, and tarsi; tarsus I with 40 distinct pseudosegments.

**Female** (***paratype***, SYNU-Ar00440): Similar to male, habitus as in Fig. 14G, H. Total length 4.71 (4.85 with clypeus), carapace 1.39 long, 1.56 wide, opisthosoma 3.32 long, 1.76 wide; tibia I: 8.15; tibia I L/d: 54. Eye interdistances and diameters: PME–PME 0.18, PME 0.14, PME–ALE 0.04, AME–AME 0.05, AME 0.08. Sternum width/length: 0.96/0.90. Clypeus brown. Epigyne posteriorly straight, with knob (kn in Fig. 14A). Vulva with nearly U-shaped, sclerotized anterior arch (aa in Fig. 14B) and pair of nearly elliptical pore plates (pp in Fig. 14B).

##### Variation.

Tibia I in two paratype males (SYNU-Ar00438–39): 10.26, 10.64. Tibia I in the other two paratype females (SYNU-Ar00441–42): 8.08, 8.33.

##### Habitat.

Underside of overhang on rocky cliffs in mountainous area.

##### Distribution.

Hebei; known only from the type locality (Figs 1, 2F).

## ﻿Discussion

This study fills a zoogeographical gap in China by collecting, for the first time, species belonging to the *Pholcusphungiformes* species group from the mountainous regions between the Yanshan-Taihang and Changbai Mountains. Nevertheless, there remains another unexplored region: North Korea. Currently, only one species, *P.parkyeonensis* Kim & Yoo, 2009, has been recorded from the southernmost part of North Korea (Kim and Yoo 2009). Given the high diversity of the *P.phungiformes* species group in the Changbai and Taebaek-Sobaek Mountains, as well as the similar landforms and habitats in neighboring North Korea, we anticipate that at least 20–30 species could be discovered if an investigation were to be conducted there. Unfortunately, it is currently difficult to conduct such an investigation in North Korea, as the country lacks experts in this field (Zhang et al. 2022; Yang et al. 2023; WSC 2025) and foreign experts face challenges in surveying there.

## Supplementary Material

XML Treatment for Pholcus

XML Treatment for Pholcus chaoyang

XML Treatment for Pholcus hebei

XML Treatment for Pholcus huludao

XML Treatment for Pholcus jinzhou

XML Treatment for Pholcus liaoning

XML Treatment for Pholcus qin

## ﻿Additional information

### Author ORCIDs

Jinglin Li https://orcid.org/0009-0008-3932-309X

Qiaoqiao He https://orcid.org/0000-0002-9381-7444

Shuqiang Li https://orcid.org/0000-0002-3290-5416

Zhiyuan Yao https://orcid.org/0000-0002-1631-0949

### Data availability

All of the data that support the findings of this study are available in the main text.
